# Does Recalibration Response Shift Alter Perceived Treatment Effects in Patient-Reported Outcomes After Anterior Cruciate Ligament Reconstruction?

**DOI:** 10.1177/23259671261463386

**Published:** 2026-08-25

**Authors:** Hana Marmura, Alan MJ Getgood, Dianne M Bryant

**Affiliations:** † Department of Exercise and Sport Science, University of North Carolina at Chapel Hill College of Arts and Sciences, Chapel Hill, Durham, North Carolina, USA; ‡ School of Physical Therapy, Western University Faculty of Health Sciences, London, Ontario, Canada; § Western University Schulich School of Medicine & Dentistry, London, Ontario, Canada; ‖ Fortius Clinic City, London, England, UK; ¶ Department of Health Research Methods, Evidence and Impact, McMaster University Faculty of Medicine, Hamilton, Ontario, Canada; Investigation performed at the Faculty of Health Sciences, Western University, and the Fowler Kennedy Sport Medicine Clinic, London, Ontario, Canada

**Keywords:** anterior cruciate ligament, knee, psychological aspects of sport, research, response shift, statistics

## Abstract

**Background:**

Patient-reported outcome measures (PROMs) are used longitudinally after anterior cruciate ligament reconstruction (ACLR). However, PROM scores depend on patients’ perspectives, which are likely to change over time, particularly after a health-related event. This phenomenon, known as recalibration or "response shift,” may limit our ability to accurately interpret PROMs.

**Purpose:**

To identify whether recalibration response shift effects are present in PROM scores after ACLR and to investigate the influence of coping styles and life events on outcomes and recalibration response shift after ACLR.

**Study Design:**

Cohort study; Level of evidence, 2.

**Methods:**

Patients having primary ACLR were recruited to complete the Knee injury and Osteoarthritis Outcome Score (KOOS) and International Knee Documentation Committee Subjective Knee Form (IKDC SKF) preoperatively and 1 and 2 years postoperatively. At both postoperative time points, patients completed a second KOOS and IKDC SKF based on their recollection of preoperative status, as well as brief COPE (Coping Orientation to Problems Experienced) and life event questionnaires. Unadjusted change (traditionally measured change), adjusted change (accounting for the patient perspective), and the magnitude of the recalibration response shift (the difference in change scores) were calculated for all outcomes, along with the proportion of patients exhibiting meaningful positive recalibration, meaningful negative recalibration, or no meaningful recalibration. Linear regression analyses evaluated the influence of coping strategies and life events on postoperative PROM scores and the magnitude of recalibration response shift.

**Results:**

Of 171 participants (mean age, 24 years, 56% male), 158 (92%) and 146 (85%) completed 1 and 2 years follow-up, respectively. Adjusted change was significantly greater than unadjusted change (*P* < .05) for all PROM scores except KOOS Sport at 1 year postoperatively (*P* = .17), with the magnitude of recalibration response shift ranging from 3 to 18 points. Three-quarters of participants’ scores indicated a meaningful shift in response in at least 1 PROM. Coping and life event variables predicted follow-up PROM scores but not the magnitude of recalibration response shift.

**Conclusion:**

A meaningful recalibration response shift was clearly identified in IKDC SKF and KOOS scores 1 and 2 years after ACLR. Traditional within-patient change scores do not reflect participants’ perceived change in constructs after ACLR, and recalibration response shifts may alter perceived treatment effects (both over- and underestimation).

Investigation of outcomes after anterior cruciate ligament (ACL) reconstruction (ACLR) is a mainstay of orthopaedic and sports medicine research. As the medical landscape becomes increasingly patient-centered, patient-reported outcome measures (PROMs) are used as the primary outcome in many studies. However, there is a notable discrepancy between subjective patient-reported outcomes and objective clinical outcomes in the results of many clinical trials and prospective cohort studies investigating ACLR-related surgical interventions. PROM scores may not reflect treatment effects observed for binary outcomes and often provide little information to influence clinical practice.^13,15,17,42^ Response shift theory provides one explanation as to why this may occur.

Patient-reported scores largely depend on respondents’ perspectives and the individual frame of reference used to interpret each question and outcome (eg, pain, function, quality of life, sport participation). Individuals’ priorities, standards, and conceptualizations of such constructs are likely to change over time, particularly following a health-related event.^35^ This phenomenon is explained by the response shift model. In clinical research, it is assumed that observed change measured by PROMs appropriately represents change in the target construct.^39^ Response shift is present when the observed change (ie, change in PROM scores) is not fully explained by the target change (ie, true patient perception of change in the construct intended to be measured).^39^ For example, a patient may show no objective improvement in sport participation over time on an outcome measure. Still, the magnitude of the improvement may be different from what the patient perceives due to adjusted expectations. If patients have vastly different perspectives when completing the same PROM at 2 time points, our confidence in interpreting true change is limited.

Proposed mechanisms by which response shift may occur include recalibration (a change in internal standards or anchors), reprioritization (a change in values), and reconceptualization (a change in how patients conceptualize target constructs, such as quality of life).^39^ If a construct is being recalibrated, reprioritized, or reconceptualized across time, patients will complete pre- and postintervention PROMs with differing frames of reference that cannot be compared with measure target change.^39^ The “then-test” is a common methodology used to identify recalibration response shift, specifically investigating the recalibration of quantitative outcome scores by having patients complete a new preintervention score at follow-up timepoints (ie, in the same frame of reference as when completing the follow-up PROMs).^34^

Response shift has been identified in the PROMs used to assess outcomes after various orthopaedic procedures and conditions, including spine conditions, ankle instability, rotator cuff repair, and hip arthroplasty.^21^ Specific to the knee, response shift has been identified in patients undergoing knee arthroplasty^23,24,41^ and microfracture.^3^

It is important to identify response shift and understand its mechanisms in specific clinical populations because evaluations of PROMs that do not account for response shift effects may result in misleading conclusions about intervention effectiveness.^2^ Surprisingly, no studies to date have investigated whether response shift is present in PROMs after ACLR. Individuals with ACL injury tend to be young, with lifestyles and environments more likely to change throughout recovery than the older populations already shown to experience response shift after orthopaedic procedures.^23,24,41^ Therefore, we hypothesized that response shift would occur in the ACL patient population. For example, patients undergoing ACLR may *recalibrate* what experiencing pain “often” means to them, which will affect how they respond to this question on a PROM at different timepoints.

The theoretical model of response shift suggests various coping strategies, social comparison, social support, goal reordering, reframing expectations, and spiritual practice as mechanisms by which the magnitude and type of response shift are influenced and mediated.^22,35,39^ It is common for patients to adopt coping mechanisms after an event such as ACL injury and reconstructive surgery. Additionally, patients experience a plethora of other changes and significant life events that may influence PROMs but are rarely asked about or accounted for. Psychosocial factors are being increasingly recognized as influential to postoperative ACLR outcomes.^7^ However, little research has been conducted on the influence of coping styles or confounding life changes after ACL injuries and reconstructions, especially in the context of response shift.

The objectives of this study were as follows: (1) identify whether recalibration response shift is present in PROM scores after ACLR, and (2) investigate the influence of coping styles and life events on PROMs and the magnitude of recalibration response shift after ACLR.

## Methods

### Participants

Patients were recruited from the clinics of four orthopaedic surgeons at the Fowler Kennedy Sport Medicine Clinic from July 2021 to February 2023. The eligibility criteria for this study were designed to reflect those commonly used in ACL injury intervention studies. Patients were eligible to participate if they were undergoing a primary ACLR, skeletally mature, could communicate in English, and were available for follow-up. Patients were not excluded based on any meniscal injuries or procedures. Patients were excluded from the study if they had an ACLR, a multiligament injury, a fracture (other than a Segond fracture), or a cartilage defect that required treatment other than debridement. Patients who did not complete preoperative questionnaires before their surgery were withdrawn as planned analyses required preoperative data. This study was approved by the Western University Health Sciences Research Ethics Board (IRB No.: 119115).

### Study Design

We conducted a prospective cohort study of patients undergoing ACLR to investigate whether a recalibration response shift was present in PROMs from pre- to 1 and 2 years postoperative using the test-retest methodology.^31,32,34^ At baseline, participants completed the Knee injury and Osteoarthritis Outcome Score (KOOS) and the International Knee Documentation Committee Subjective Knee Form (IKDC SKF). The KOOS and IKDC SKF questionnaires were chosen because they are the 2 most commonly used PROMs for assessing outcomes after ACL injury and reconstruction.^36^

The KOOS is a knee-specific PROM consisting of 42 items across 5 separately scored domains: Pain, Other Symptoms, Function in Daily Living, Function in Sports/Recreation, and Knee-Related Quality of Life.^26^ The IKDC SKF is a knee-specific 18-item questionnaire querying symptoms, function, and sports activities.^27^ The KOOS scales and IKDC SKF are scored from 0 to 100 (worst to best). Both outcome measures have been validated and are used across multiple ACL registries and clinical trials.

One and 2 years after ACLR surgery, participants completed the KOOS, IKDC SKF, patient-acceptable symptom state (PASS) question, Life Events, and brief-COPE (Coping Orientation to Problems Experienced) questionnaires according to their postoperative status. The PASS is a single question asking, “Taking into account all the activity you have during your daily life, your level of pain, and also your activity limitations and participation restrictions, do you consider the current state of your knee satisfactory?” with a binary yes or no response. The brief-COPE is a validated questionnaire used to assess individuals’ coping styles.^8^ The questionnaire consists of 28 items, with 14 scales measuring 14 different coping strategies.^8^ Each coping strategy is scored from 2 points (representing no use of the strategy) to 8 points (representing high use of the strategy) and is defined by 2 items (each scored from 1 “I haven't been doing this at all” to 4 “I’ve been doing this a lot”).^8^ Additionally we calculated a positive coping style score by summing the scores for the following 10 strategies: active coping, emotional support, humor, instrumental support, planning, positive reframing, religion, self-distraction, and venting (ranging from 20 to 80 points)^8^ and a negative coping style score by summing the scores of the remaining 4 strategies: denial, disengagement, self-blame, and substance use (ranging from 8 to 32 points) based on previous literature.^8^ The life events questionnaire is a 23-item questionnaire our research team created to understand the magnitude of change to a patient's lifestyle and the occurrence of significant events (Supplemental File 1). The first question asks participants, “How has your lifestyle changed since your ACL injury (not necessarily because of your injury)?” with the options akin to the global rating of change scale: multiple significant changes; a few significant changes; some changes, not that significant; small changes, not significant; no changes. The questionnaire then includes 21 life events related to sport, school/work, family/loved ones, and health (ie, stopped playing a sport, graduated from a school program, experienced a mental health issue). For each event, participants select whether it had occurred since their ACL injury (yes/no). Lastly, there is an item where participants can report and describe any life event not listed that they have experienced since the injury. Any ACLR-related adverse events and resultant reoperations were recorded at postoperative follow-up.

Importantly, at 1 and 2 years postoperative, participants completed their follow-up outcome measures and then completed a “then-test” version of both the KOOS and IKDC SKF, which asked participants to complete the questionnaires based on their preoperative status.^31,32,34^ The then-test provides an alternative set of preoperative PROM scores, which may be better comparators to postoperative scores because both are completed by participants at the same time (ie, with the same perspective and frame of reference). Therefore, if patients have recalibrated what constructs/scores/response options mean to them, this will be reflected in the recalled pre- and postoperative scores, but not the actual preoperative scores completed 1 or 2 years earlier. The 1-year postoperative time point is a traditional follow-up time point, when patients often make important judgments about their recovery and decisions regarding return to sport and physical activity. The 2-year postoperative time point represents the minimum acceptable follow-up time point to understand adverse events and rerupture rates in these patients. Patients completed all outcome measures at all timepoints regardless of outcome.

### Statistical Analyses

#### Identifying Recalibration Response Shift

The previously established then-test methodology^31,32,34^ was used to determine whether a recalibration response shift was occurring after ACLR. Actual preoperative (completed preoperatively) scores were compared to recalled preoperative (completed 1 or 2 years postoperatively) scores to understand how utilizing each version might affect the interpretation of improvement in PROMs after ACLR. The magnitude of agreement/association between the actual and recalled preoperative scores was first estimated by calculating an intraclass correlation coefficient (ICC) and its 95% CI for each of the 5 KOOS subscale scores and the overall IKDC SKF score.

As per then-test methodology, the following formulas were used to operationalize recalibration response shift in each PROM (reported as means and 95% CIs)^24^:

1. Unadjusted change = postoperative score – actual preoperative score
2. Adjusted change = postoperative score – recalled preoperative score
3. Recalibration response shift = adjusted change – unadjusted change.

Notably, the unadjusted change scores represent values that would be traditionally reported in all preoperative-postoperative studies. Adjusted change scores represent change using the same frame of reference to assess pre- and postoperative status. The then-test methodology investigates the recalibration mechanism of response shift by comparing change scores using 2 different preoperative anchors. If respondents’ internal standards of measurement have changed, adjusted and unadjusted change scores will be different, suggesting a recalibration response shift.^39^ If internal standards are consistent over time, the 2 change scores will be similar, indicating no meaningful recalibration. The magnitude of the recalibration response shift is calculated as the difference between these 2 change scores and is also equivalent to the difference between actual and recalled preoperative scores.

Paired *t* tests were conducted to compare actual preoperative, postoperative, and recalled preoperative means, and to compare unadjusted and adjusted mean change scores. The significance level was set at *P* < .05.

#### Understanding Recalibration Response Shift

Recalibration can occur in both positive and negative directions, indicating different patient perspectives and psychological processes.^24^ Positive recalibration occurs when participants underestimate their previous status and perceive larger improvements.^24^ Negative recalibration occurs when participants overestimate their previous status and perceive smaller improvements.^24^ Some degree of recalibration is always expected, as it is unlikely that patients will recall and score their preoperative status across multiple questionnaire items exactly as they did 1 or 2 years prior. As such, not all recalibrations are meaningful for interpreting outcomes or for clinical practice. It has been suggested to use a threshold to identify when a significant amount of recalibration has occurred.^24^

There is a range of proposed minimal clinically important differences and minimal detectable changes for the KOOS subscales and IKDC SKF, with no agreed-upon value specifically for the post-ACLR population.^14,37^ Therefore, a threshold of 10 points was chosen to indicate meaningful recalibration based on reported minimally important change values and an estimated minimally important difference using a moderate effect size (0.5) and a conservative standard deviation estimate of 20 points for the KOOS and IKDC SKF.^14,19,20,25^ Therefore, recalibration response shifts of magnitudes from −10 to +10 points were not considered to represent an important amount of recalibration, and shifts >10 points in either direction were considered clinically meaningful.

Three groups were created for each PROM score: meaningful positive recalibration (adjusted change > unadjusted change by ≥10 points), meaningful negative recalibration (adjusted change < unadjusted change by ≥10 points), and no meaningful recalibration (adjusted change and unadjusted change are within 10 points of each other). The proportion of patients in each group was reported for each outcome at 1 and 2 years postoperatively, as well as the proportion of patients with meaningful recalibration in either direction (positive or negative).

Descriptive statistics were used to describe coping strategies and life events reported among participants. Linear regression analyses were conducted to understand whether coping styles and life changes/events might influence postoperative scores and the magnitude of recalibration response shift identified in patient change scores. A multivariable regression model was created with the following *a priori* predictors: age, sex, adverse events, PASS, magnitude of lifestyle change (rated on a 5-point scale from no changes to multiple significant changes), number of life events occurring in the first postoperative year, negative coping style score, and positive coping style score. These models were tested to predict 1- and 2-year follow-up scores and the magnitude of the recalibration response shift for each PROM at each time point. Significant predictors (*P* < .05) were retained in the final models. Visual inspection of scatter plots and quantile-quantile plots was used to assess assumptions of normality, linearity, and homogeneity of variance. Multicollinearity was assessed based on the variance inflation factor (VIF) (ensuring no predictors with VIF >10), and the Cook distance was used to identify influential points.

### Sample Size

A required sample size of 132 patients was calculated for this study based on the number of participants needed to estimate the agreement (ICC) between actual and recalled preop scores.^5^ The ICC was estimated at 0.75, using expected values of between-subject (20 points) and within-subject (10 points) variance in outcome scores, with a 95% CI.^4,9,18,20,25^ Accounting for 15% attrition across the 2-year follow-up period, we aimed to recruit a total of 155 participants.

## Results

### Participants

A total of 200 patients were screened, of whom 171 were eligible to be included in the analysis (7 were ineligible based on previous ACL injury/surgery or concomitant injury, and 21 withdrew based on lack of preoperative data). The patients included in this study were predominantly young (mean age, 24 years); their ages ranged from 13 to 58 years. Patient characteristics are provided in Table 1. A total of 158 participants completed a 1-year follow-up (92%), and 146 participants completed a 2-year follow-up (85%).

**Table 1:** Patient Characteristics, PASS, and Adverse Events Reported at 1 and 2 years After ACLR*^*a*^*

Characteristic N = 171	Mean (SD) or N (%)	
Age at time of surgery, years	24 (9)	
Sex		
Male	97 (57)	
Female	74 (43)	
	1 Year Postop n = 158	2 Years Postop n = 146
PASS		
Yes	108 (68)	117 (80)
No	50 (32)	25 (17)
Missing	0 (0)	4 (3)
Adverse Events	18 (11)	13 (9)
Graft failure	2 (1)	2 (1)
Contralateral ACL tear	0 (0)	1 (1)
Meniscal tear	4 (3)	5 (3)
Cyclops lesion	6 (4)	0 (0)
Increased pain/hardware irritation	5 (3)	4 (3)
Arthrofibrosis	1 (1)	0 (0)
Deep MCL sprain	0 (0)	1 (1)
Reoperation, any	14 (9)	8 (5)

a ACL, anterior cruciate ligament; ACLR, ACL reconstruction; MCL, medial collateral ligament; PASS, patient acceptable symptom state; Postop, postoperatively; SD, standard deviation.

At 1 and 2 years postoperatively, 68% (107/158) and 80% (117/146) of participants reported that they considered the current state of their knee satisfactory based on the PASS score. A total of 18 participants (11%) reported adverse events, with 14 requiring a reoperation (9%) in the first postoperative year. In the second postoperative year, 13 participants (9%) reported adverse events, with 8 requiring a reoperation (5%) (Table 1).

### Identifying Recalibration Response Shift

**Figure 1.**
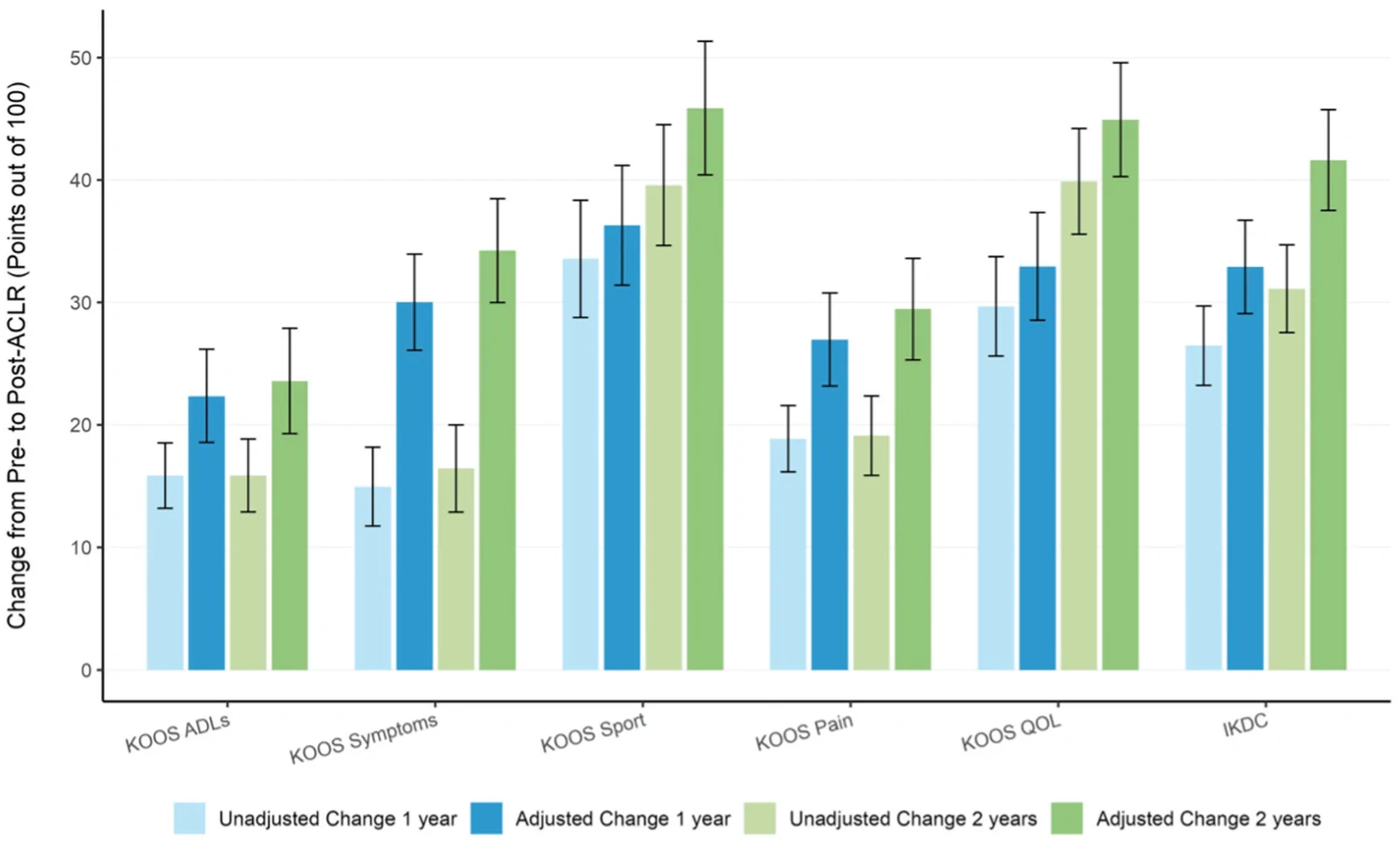
Mean unadjusted and adjusted change scores in patient-reported outcome measure scores 1 and 2 years after ACLR. Error bars represent 95% CIs. Adjusted change was significantly greater than unadjusted change (*P* < .05) for all scores at both time points except for KOOS Sport at 1-year postoperative. ACLR, anterior cruciate ligament reconstruction; ADLs, Activities of daily living; IKDC SKF, International Knee Documentation Committee Subjective Knee Form; KOOS, Knee injury and Osteoarthritis Outcome Score; QoL: Quality of life.

Only moderate agreement was seen between baseline actual and recalled preoperative scores. The strength of agreement (ICCs) ranged from 0.27 (95% CI, –0.01 to 0.49) for KOOS Symptoms at 2 years postoperative to 0.58 (95% CI 0.43 to 0.69) for IKDC SKF at 1 year postoperative, with patients generally recalling their preoperative status as worse than the actual values recorded at baseline (general trend of positive recalibration). Preoperative, recalled preoperative, and postoperative (1 and 2 years) IKDC SKF and KOOS scores are displayed in Table 2. As expected, mean postoperative scores were significantly higher than actual or recalled preoperative scores for all outcomes (*P* < .05), indicating improvement over time. Notably, actual and recalled preoperative scores were significantly different for all outcomes (*P* < .05) except for the KOOS Sport score at 1 year (*P* = .17). Therefore, adjusted change was significantly greater than unadjusted change (*P* < .05) for all PROMs except KOOS Sport at 1 year (*P* = .17) (Figure 1), with a magnitude of recalibration response shift ranging from 3 to 18 points (Table 2).

**Table 2:** Preop, Recalled Preop, and Postop PRO Scores at 1 and 2 Years After ACLR*^*a*^*

	Preop	Recalled Preop, 1 Year Postop	Recalled Preop, 2 Years Postop	1 Year Postop	2 Years Postop	Magnitude of Recalibration Response Shift, 1 Year Postop	Magnitude of Recalibration Response Shift, 2 Years Postop
KOOS							
ADLs	78.7 ± 17.1	**72.4 ± 24.7**	**71.4 ± 22.8**	**94.7 ± 9.6**	**95.0 ± 11.9**	6.5 (3.4 to 9.7)	7.9 (4.7 ± 11.2)
Pain	68.9 ± 17.1	**60.7 ± 23.1**	**58.9 ± 20.2**	**87.6 ± 11.9**	**88.3 ± 15.7**	8.2 (5.1 to 11.4)	10.3 (7.2 to 13.5)
Symptoms	64.4 ± 17.7	**49.2 ± 22.4**	**47.3 ± 20.0**	**79.3 ± 16.1**	**81.5 ± 15.8**	15.3 (12.0 to 18.7)	17.8 (14.4 to 21.2)
Sport	40.7 ± 24.4	38.5 ± 25.7	**34.2 ± 23.2**	**74.7 ± 22.7**	**80.1 ± 23.0**	2.9 (–1.3 to 7.0)	6.4 (2.4 to 10.4)
QoL	30.2 ± 16.9	**27.2 ± 20.2**	**25.4 ± 18.2**	**60.0 ± 24.8**	**70.4 ± 23.8**	3.3 (0.4 to 6.2)	5.1 (2.1 to 9.2)
IKDC SKF	51.4 ± 16.4	**45.5 ± 20.6**	**41.6 ± 18.0**	**78.3 ± 16.0**	**83.4 ± 16.4**	6.3 (3.7 to 8.9)	10.2 (7.7 to 12.8)

a Data are reported as mean ± SD and mean (95% CI). Boldface values indicate mean values that are significantly different from the preoperative values reported for that PROM (*P* < .05). ACLR, anterior cruciate ligament reconstruction; ADLs: Activities of daily living; IKDC SKF, International Knee Documentation Committee Subjective Knee Form; KOOS, Knee injury and Osteoarthritis Outcome Score; Postop, postoperative; Preop, preoperative; PRO, patient-reported outcome; PROM, patient-reported outcome measure; QoL, quality of life.

### Understanding Recalibration Response Shift

At 1 and 2 years postoperative, across all PROMs, 52% to 78% of patients’ scores indicated meaningful recalibration response shift, meaning pre- to postoperative change was over- or underestimated by at least 10 points using traditional measures (Table 3). Positive recalibration (ie, perceiving a greater change than what would have been documented by a traditional preoperative-postoperative change score) was more prevalent (32% to 69% of patients) than negative recalibration (ie, perceiving a smaller change than what would have been documented by a traditional preoperative-postoperative score, 13% to 27% of patients).

**Table 3:** Proportion of Patients With Positive, Negative, or No Meaningful Recalibration Response Shift in PROMs at 1 and 2 Years After ACLR*^*a*^*

	Positive Recalibration >10 Points		Negative Recalibration < –10 Points		Meaningful Recalibration, Either Direction	
	1-Year Postop, n = 158	2-Year Postop, n = 146	1-Year Postop, n = 158	2-Year Postop, n = 146	1-Year Postop, n = 158	2-Year Postop, n = 146
KOOS						
ADLs	56 (35)	60 (41)	26 (16)	23 (16)	82 (52)	83 (57)
Pain	73 (46)	68 (47)	27 (17)	15 (10)	100 (63)	83 (57)
Symptoms	98 (62)	100 (68)	21 (13)	13 (9)	119 (75)	113 (77)
Sport	51 (32)	57 (39)	43 (27)	29 (20)	94 (59)	86 (59)
QoL	62 (39)	58 (40)	29 (18)	23 (16)	91 (58)	81 (56)
IKDC SKF	67 (42)	71 (49)	23 (15)	9 (6)	90 (57)	80 (55)

a Data are presented as n (%). ACLR, anterior cruciate ligament reconstruction; ADLs: Activities of daily living; IKDC SKF, International Knee Documentation Committee Subjective Knee Form; KOOS, Knee injury and Osteoarthritis Outcome Score; Postop, postoperative; Preop, preoperative; PROM, patient-reported outcome measure; QoL, quality of life.

The KOOS Symptoms score showed the greatest recalibration at both time points. The KOOS Sport score had the most balanced distribution of positive and negative recalibration and the largest proportion of negative recalibration compared with the other PROMs, which explains why overall adjusted and unadjusted change scores appeared more similar in this outcome (ie, the positive and negative direction of recalibration cancel each other out in mean change scores).

### Coping Styles and Life Changes

Patients tended to report positive/adaptive coping styles more often than negative/maladaptive coping styles. The coping styles patients reported using most frequently as measured by the brief-COPE questionnaire (score range: 2 to 8 points) were acceptance (1y: 5.8 ± 1.8, 2y: 5.4 ± 1.9), active coping (1y: 5.2 ± 1.9, 2y: 4.5 ± 1.9), planning (1y: 4.7 ± 1.8, 2y: 4.2 ± 1.9), positive reframing (1y: 4.4 ± 1.8, 2y: 4.2 ± 1.9), and self-distraction (1y: 4.4 ± 1.8, 2y: 4 ± 1.7), whereas disengagement (1y: 2.6 ± 1.1, 2y: 2.4 ± 1), denial (1y: 2.4 ± 1.1, 2y: 2.4 ± 1), and substances (1y: 2.4 ± 0.9, 2y: 2.3 ± 1) were least common.

**Figure 2.**
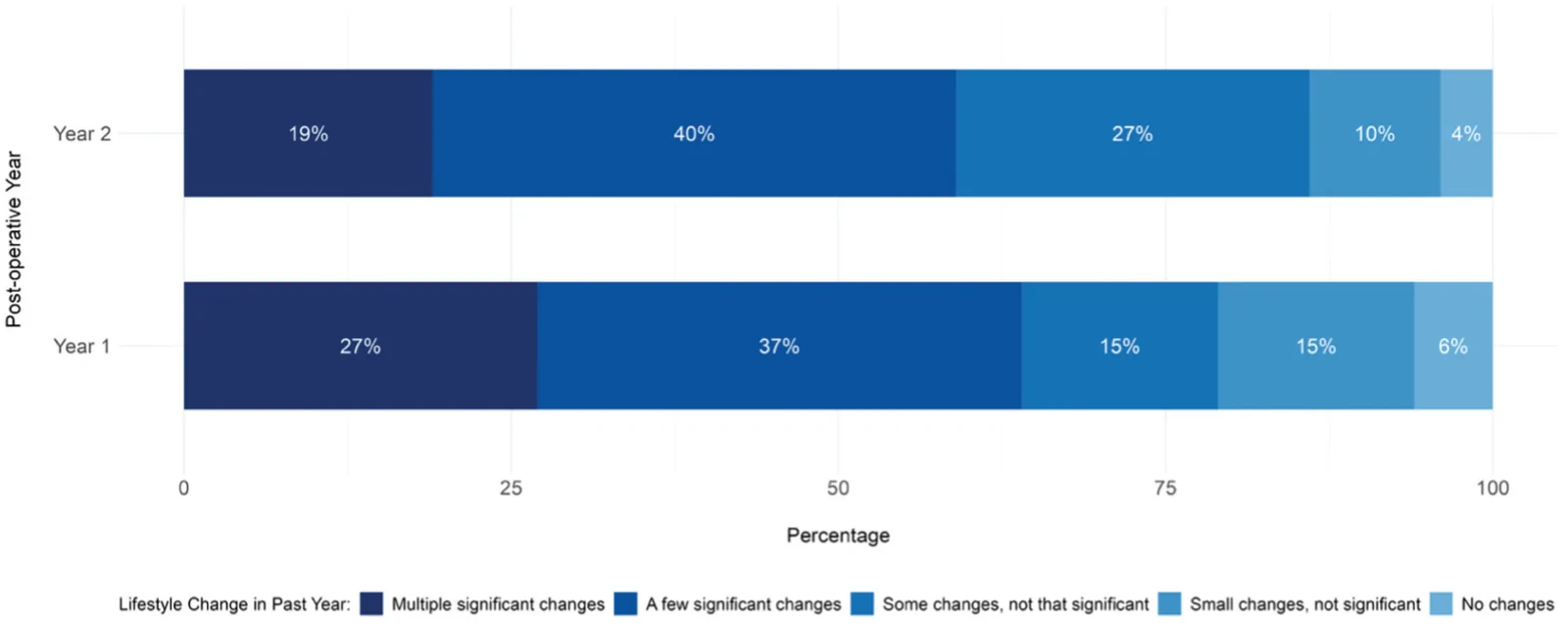
Magnitude of lifestyle change reported by patients in the first and second postoperative year after anterior cruciate ligament reconstruction.

Over half of patients experienced at least a few significant life changes in postoperative years 1 and 2 (64% and 59%, respectively; Figure 2). Life events reported by at least 10% of participants are shown in Table 4.

**Table 4:** Life Events Reported by at Least 10% of Patients 1 and 2 Years After ACLR*^*a*^*

Life Event	1-Year Postop n = 158	2-Year Postop, n = 146
Stopped participating in a sport	122 (77)	88 (60)
Started a new job	81 (51)	86 (59)
Started a new school/education/training program	50 (32)	65 (45)
Quit or terminated from a job	48 (30)	38 (26)
Graduated from high school, college, university, or any other program	47 (30)	76 (52)
Experienced the death of a loved one	39 (25)	27 (18)
A loved one experienced a serious physical health issue, illness, or trauma	30 (19)	33 (23)
Started my first "real" job	29 (18)	36 (25)
Started participating in a new sport	26 (16)	38 (26)
Relocated to a new city, province, or country	24 (15)	22 (15)
I suffered a serious mental health issue or trauma	19 (12)	16 (11)
A loved one experienced a serious mental health issue or trauma	18 (11)	14 (10)

a Data are presented as n (%). ACLR, anterior cruciate ligament reconstruction; Postop, postoperative.

Because assumptions of normality were violated in regression models predicting individual KOOS subscale scores, linear regression analyses were conducted to predict composite KOOS scores (ie, the total score across all KOOS subscales) and total IKDC SKF scores. Statistically significant predictors of postoperative PROM scores were consistent for KOOS and IKDC SKF scores. They included age, experiencing an adverse event, the magnitude of lifestyle change reported, negative coping style score, and PASS (Table 5). The final linear regression models explained 52% to 64% of the variance in postoperative composite KOOS and total IKDC SKF scores (Table 5).

**Table 5:** Final Predictive Models for PROMs 1 Year After ACLR Surgery*^*a*^*

	Composite KOOS Score		IKDC SKF Score	
	1-Year Postop	2-Year Postop	1-Year Postop	2-Year Postop
Age	–0.27 (–0.43 to −0.09)	-	–0.27 (–0.47 to −0.08)	-
Adverse event	–9.67 (–14.86 to −4.46)	–16.02 (–21.98 to −10.05)	–6.60 (–12.54 to −0.66)	–13.31 (–19.28 to −7.34)
Lifestyle change	–3.02 (–4.38 to −1.64)	–2.77 (–4.43 to −1.12)	–2.79 (–4.36 to −1.22)	–3.04 (–4.70 to −1.39)
Negative coping	–1.27 (–1.95 to −0.59)	–1.43 (–2.20 to −0.66)	–1.69 (–2.46 to −0.91)	–1.42 (–2.19 to −0.64)
PASS	13.29 (9.67 to 16.91)	20.49 (15.43 to 25.55)	14.20 (10.06 to 18.34)	23.96 (18.9 to 29.0)
Adjusted *R*^2^	0.58	0.61	0.52	0.64

a Results are reported as coefficients, representing the change in the outcome score resulting from a 1-unit increase in the predictor variable, with associated 95% CIs. All variables presented are significant predictors of the outcome with *P* < .05. ACLR, anterior cruciate ligament reconstruction; IKDC SKF, International Knee Documentation Committee Subjective Knee Form; KOOS, Knee injury and Osteoarthritis Outcome Score; PASS, patient acceptable symptom scale; Postop, postoperative; PROM, patient-reported outcome measure.

Regression models predicting the magnitude of recalibration response shift in composite KOOS and total IKDC SKF scores were much less accurate, explaining between 2% and 29% of the variance in the magnitude of recalibration response shift. The only significant predictors of the magnitude of recalibration response shift in PROM scores were experiencing an adverse event (IKDC SKF at 1y postop: –9.34 [95% CI, –17.68 to −1.59]; Composite KOOS at 2y postop: β = 22.05 [95% CI, 12.49 to 31.62]) and achieving a PASS (Composite KOOS at 1y postop: β = −13.83 [95% CI = −19.47 to −8.19]), with inconsistent associations across PROMs and timepoints. Coping and life event variables were not strong predictors of the magnitude of recalibration response shift 1 or 2 years after ACLR.

## Discussion

### Identifying Recalibration Response Shift

The main finding of this study was the clear identification of recalibration response shift occurring in patients completing the KOOS and IKDC SKF PROMs 1 and 2 years after ACLR. Three-quarters of patients displayed a recalibration response shift of 10 points or more (ie, at least 10% under- or overestimation of PROM scores out of 100 points) in at least 1 PROM at both 1- and 2-year follow-up timepoints. This means that 75% of participant improvements (preoperative-postoperative treatment effects) would have been meaningfully under- or overestimated in a traditional preoperative-postoperative study design. Participants are likely to use recalled preoperative status and/or perceived improvement as anchors or data points to appraise their current/postoperative status. Therefore, within-patient longitudinal change scores traditionally relied on clinical research may be unreliable measures of true change at the individual level. Because recalibration occurs in both the positive and negative directions, understanding the impact of response shift at the group level may be more difficult.

### Understanding Recalibration Response Shift

Both positive and negative shifts in recalibration responses were observed in our study sample. However, positive recalibration was more common (35% to 69% of patients) than negative recalibration (9% to 27% of patients). This mirrors the trend reported in a similar study involving patients after total knee arthroplasty, in which 27% and 9% of participants exhibited positive and negative recalibration in PROMs, respectively.^24^ As hypothesized, a significantly higher proportion of patients undergoing ACLR exhibited meaningful recalibration response shift when compared with the total knee arthroplasty sample: 52% to 78% of the ACLR patients in the present study compared with 36% of the knee arthroplasty sample.^24^ Our study extends recalibration response shift literature in orthopaedics to include an ACL patient population and reiterates that traditional assessment and interpretation of change may lead to inaccurate conclusions regarding individual outcomes and treatment effectiveness.^2,21,28^

The highest proportion of positive recalibration was observed in KOOS Symptoms scores (69%), whereas the most negative recalibration was observed in KOOS Sport scores (27%). One potential explanation for this could be the patient status for each domain/construct at the 1- and 2-year postoperative time points. For example, most patients will have minimal symptoms described in the KOOS Symptoms scale (stiffness, swelling, catching/grinding/clicking, and loss of range of motion) after the first postoperative year. Therefore, patients are likely to perceive a substantial improvement in symptoms and may judge their baseline symptoms as worse than they were, thereby creating this larger subjective change. In contrast, many participants will be just starting to return to sport at 1 year postoperative. They may still be encountering difficulty with many of the activities included on the KOOS Sport score (running, jumping, squatting, twisting/pivoting) even at 2 years postoperative, especially when comparing themselves to the preinjury level of sport participation and performance. Therefore, patients may perceive a smaller improvement in this domain and recall their baseline sport scores more similarly to or better than before, reflecting this smaller change.

This study introduced new variables not traditionally measured in ACLR studies: coping styles and significant life events. We hypothesized that these variables would be influential to reported PROM scores and are also important to understand for optimal clinical practice and individualized treatment.^7^ Notably, over half of patients reported experiencing a few or multiple significant life changes within 2 years of ACLR surgery. Between 30% and 60% of patients reported some change in school program or job, and a quarter reported losing a loved one in the first postoperative year. These events are all likely to influence patients’ priorities and perspectives on various constructs that are measured by PROMs^16^ and may also affect access to rehabilitation resources and/or opportunities for desired activities and sport participation. Reflecting this idea, a greater magnitude of lifestyle change reported on the Life Events questionnaire was a significant predictor of worse postoperative KOOS and IKDC SKF scores in the present study.

It is proposed that a response shift may result from cognitive and/or behavioral coping strategies used to manage negative situations or emotions.^39^ Previous studies of patients undergoing sport-related knee surgeries indicated that positive reframing,^12^ problem-focused strategies,^11^ and the use of instrumental support^11^ were associated with improved outcomes. Higher scores for negative coping style were significantly associated with worse postoperative outcomes in our sample of ACLR patients, while positive coping style scores were not significantly associated with postoperative outcomes. We hypothesized that coping and life events would be important predictors of both follow-up PROM scores and the magnitude of the recalibration response shift; however, only the former appears to be true. Larger lifestyle changes, adverse events, and higher negative coping style scores were significant predictors of worse postoperative PROM scores. Contrary to our hypotheses, coping style and life event variables were not strong predictors of the magnitude of the recalibration response shift at 1- or 2-year postoperative periods. The prediction models for follow-up PROMs themselves were moderately accurate, explaining over half of the variance in scores. However, we were unable to consistently or accurately explain the variance in recalibration response shift values.

The nature of then-test methodology inherently raises questions regarding the role of recall bias in interpreting results. Interpreting the difference between adjusted and unadjusted change scores fully as recalibration response shift assumes patients accurately recall their preintervention status when completing the then test.^34^ As such, it has been argued that the then-test is not an optimal method of detecting recalibration response shift because discrepancies in change scores do not solely reflect recalibration but also recall bias and other health-related or cognitive processes.^29,30,33^ Unfortunately, no valid method has been established to disaggregate these processes to understand the nuance in changing perspectives. Therefore, we believe that investigating shifts in recalibration responses in ACLR patients using this method remains a worthwhile endeavor and an important contribution to the literature on how human cognitive processes may complicate the interpretation of outcomes. However, we must acknowledge that the values labeled “recalibration response shift” likely reflect a combination of changes in patient perspectives (ie, response shift) and some degree of recall error. To account for potential “noise” from inaccurate recall, we set a 10-point threshold for “meaningful recalibration,” ensuring that shifts were at least 10% of the overall scale before labeling discrepancies as recalibration response shifts. Lastly, the primary studies citing concerns about distinguishing recalibration from other sources of variance/error have been conducted in older adults (mean ages ranging from 45 to 71) with conditions that may be more likely to affect cognition and memory.^1,30,33^ These concerns are still relevant for the present study, but may hold less weight in a population of younger patients undergoing ACLR.

Further, our research team has previously conducted a nested qualitative study that identified patients’ reconceptualization of what quality of life (QoL) meant to them and the reprioritization of sport as mechanisms by which response shifts in perceived QoL scores occurred, refuting recall bias as the main contributor to the response shift phenomenon.^16^ Patients in this study were shown their actual preoperative scores after reporting their recalled preoperative scores and were asked to discuss any discrepancies (ie, response shift). Rather than reporting issues with remembering preoperative status or deferring to actual preoperative scores as the “truth” once they were revealed, patients discussed how their frame of reference had changed after the ACL injury, surgery, and recovery process, and after reflecting on new comparators and experiences to draw on when reporting outcomes. We identified the response-shift mechanisms of both reprioritization and reconceptualization (ie, the 2 mechanisms not explored in the present study) through thematic analysis of qualitative data. We proposed the SPARQ ACL Model of Sport Prioritization and Athlete Reconceptualization of Quality of Life to help frame the process of response shift among young athletes as their perspectives change after ACLR.^16^

### Implications

The results of the present study have important implications for clinical research methodology. Ignoring the possibility of response shift in an individual, sample, or patient population assumes that patient perceptions of various constructs have not changed over time and/or that patients have not adapted to their situation. Overall, this work calls into question the utility of traditional longitudinal change scores (ie, pre- to postintervention) for PROMs after ACLR and emphasizes the need to consider and account for recalibration response shift effects in clinical research. The results of this study suggest that recalibration response shift primarily affects within-patient longitudinal change, in which patients’ adaptability, coping, and evolving internal standards/comparators complicate the interpretation of improvement over time.

This is especially true for injuries and conditions with lengthy recovery periods, such as ACLR. Response shift is more likely to occur with a longer duration between PROM collections, such as the 1- and 2-year follow-ups used in this study. For shorter duration studies or measures of change, there may be less concern. For example, a previous randomized trial involving >300 patients reported excellent agreement between preoperative and 2-week PROM scores in patients undergoing ACLR or knee arthroscopy.^6^ Importantly, the results of this study do not indicate that PROMs should be completely discarded, as PROMs remain highly valuable for understanding patients’ subjective experiences of outcomes we cannot measure directly and for between-group comparisons across treatments or cohorts.

Therefore, the “so what” message of this study for clinical research moving forward is that traditional within-individual change scores do not adequately represent patient-reported outcomes after ACLR and may not be appropriate for appraising individual outcomes or within-group improvement. Instead, follow-up or postoperative scores should be used for between-group comparisons of PROMs, controlling for baseline/pre-operative PROM scores as covariates, where the association between the pre- and postoperative scores influences the precision of the postoperative comparison. We found similar weak but significant associations between actual pre- to postoperative (*r* = 0.18 to 0.34) versus recall preoperative to postoperative scores (*r* = 0.18 to 0.24). This message aligns with previous methodological work, which consistently recommends using postintervention scores adjusted for baseline values (eg, via analysis of covariance) rather than change scores.^10,38,40^

Our regression analysis indicated that the largest contributors to postoperative PROM scores were whether a patient experienced an adverse event and whether they had an acceptable symptom state. As such, studies with low adverse event rates may be unable to detect differences between groups using PROMs, and a recalibration response shift could exacerbate this issue if patients adapt or change their standards to the point where they report an acceptable symptom state despite an adverse event. Therefore, comparative intervention studies must be adequately powered to detect differences in other patient-important outcomes, such as graft failure, return to sport, et cetera, to ensure that effective interventions are not dismissed and that ineffective interventions are not promoted based solely on a lack of statistically significant differences between groups in postintervention PROMs. Given that adverse events and satisfactory outcomes may be attributable to the intervention provided, it appears likely that response shift may be partially explained by the magnitude of an intervention's treatment effect, in addition to or instead of changes in individuals’ perceptions over time.

While no established methods to account for or detect recalibration response shift have been developed, potential avenues to explore might include investigating between-group comparisons of the magnitude of recalibration response shifts across interventions, revealing preoperative scores to patients at follow-up to anchor responses, or developing PROMs with lower susceptibility to changing perspectives/frames of reference over time (eg, asking can you walk for 10 minutes without pain, versus how much pain do you have walking for 10 minutes?).

### Strengths and Limitations

This is the first study to investigate recalibration response-shift effects in PROMs after ACLR in an adequately powered prospective cohort. It is also one of a few that use a meaningful threshold for the then-test to reduce the likelihood that the observation of recalibration response shift could be attributed to random error.

Critically, this study only evaluated the recalibration mechanism for response shift, and the results are likely due to a combination of patients’ recalibration of a scale/change in internal standards for a given construct, as well as recall error. The life events questionnaire used in the study was created for this project and has not been validated, meaning scores should be interpreted with caution. However, the questionnaire mainly involves binary reporting of whether an event occurred in the past year for a patient, which is likely to be reported reliably by patients and cannot be validated against a gold-standard measure unless patients were more closely monitored over the 2 postoperative years.

## Conclusion

Meaningful recalibration, resulting in a significant shift in response, was clearly identified in IKDC, SKF, and KOOS scores at 1 and 2 years after ACLR. Positive recalibration was more common than negative recalibration; however, both processes were evident within patient responses. Traditional within-patient change scores do not reflect participants’ perceived change in important constructs after ACLR, and recalibration response shift may alter perceived treatment effects in both directions (over- and under-estimation).
